# Multimodal Imaging of a Giant Ovarian Mature Cystic Teratoma Featuring the Floating Ball Sign: A Case Report

**DOI:** 10.2174/0115734056459131260519061244

**Published:** 2026-07-01

**Authors:** Jing Chen, Na Su, Congwei Jia, Dan Wang, Huadan Xue, Yonglan He

**Affiliations:** 1 Department of Radiology, Peking Union Medical College Hospital, Chinese Academy of Medical Sciences and Peking Union Medical College, Beijing, People’s Republic of China; 2 Department of Ultrasound, Peking Union Medical College Hospital, Chinese Academy of Medical Sciences and Peking Union Medical College, Beijing, People’s Republic of China; 3 Department of Pathology, Peking Union Medical College Hospital, Chinese Academy of Medical Sciences and Peking Union Medical College, Beijing, People’s Republic of China; 4 Department of Obstetrics and Gynecology, Peking Union Medical College Hospital, Chinese Academy of Medical Sciences and Peking Union Medical College, Beijing, People’s Republic of China

**Keywords:** Ovarian mature cystic teratoma, Floating ball sign, Computed tomography, Magnetic resonance imaging, Abdominal distension, Floating ball sign

## Abstract

**Background:**

Ovarian Mature Cystic Teratomas (MCTs), the most common type of ovarian teratoma, contain tissues from all three germ layers. The “floating ball sign,” which consists of mobile spherules of sebum and keratin, is a characteristic feature seen on Computed Tomography (CT) and Magnetic Resonance Imaging (MRI). However, the imaging features can overlap with other cystic lesions, leading to potential misdiagnosis. This case report focuses on a giant MCT initially misdiagnosed as hydatid disease on CT. Its value lies in highlighting the diagnostic pitfall of over-relying on CT and demonstrating how a systematic, multimodal approach resolves uncertainty.

**Case Presentation:**

A 40-year-old female presented to our hospital due to a 3-month history of progressive, painless abdominal distension and recent urinary frequency/urgency. On physical examination, a soft, non-tender mass was palpated in the umbilical region. Initial abdominal-pelvic contrast-enhanced CT revealed a huge cystic lesion with multiple internal cystic structures resembling “daughter cysts”, a feature suggestive of hydatid disease. However, preoperative parasite tests returned negative, creating a conflict with this initial suspicion, and the mass effect caused by the lesion obscured its origin on CT. This discrepancy prompted further evaluation, which revealed mobile intracystic spherical structures via targeted ultrasound, confirming their mobility and floatability. Meanwhile, MRI identified characteristic fat content through fat-saturated sequences. These findings confirm the “floating ball sign,” which collectively supported the possible diagnosis of MCT. The diagnosis was confirmed by exploratory laparotomy and subsequent histopathological analysis.

**Conclusion:**

This case illustrates that while CT is useful for initial evaluation of giant abdominopelvic masses, its findings can be misleading. When imaging and clinical data conflict, a deliberate diagnostic reconsideration pathway using targeted ultrasound and MRI is key to avoiding misdiagnosis.

## 1 BACKGROUND/INTRODUCTION

Ovarian teratomas, which originate from pluripotent germ cells, account for a substantial proportion of all ovarian neoplasms. As the most common subtype, Mature Cystic Teratomas (MCTs) are characterized by well-differentiated tissues derived from all three germ layers: ectoderm (keratinized squamous epithelium, hair follicles), mesoderm (adipose tissue, cartilage), and endoderm (respiratory-type epithelium) [1, 2]. The “floating ball sign,” first described by Muramatsu *et al.* in 1991, is a distinctive radiological feature of MCTs, manifesting as mobile, spherical structures suspended within the cystic lumen [3]. The clinical relevance of this finding was recently underscored in a New England Journal of Medicine case report of an MCT with torsion that also featured the floating ball sign [4]. Composed primarily of sebaceous material, keratin, and hair, these spherules are visible on Computed Tomography (CT) and Magnetic Resonance Imaging (MRI). The floating ball sign has a reported prevalence of approximately 11-25% in MCTs [5, 6].

The formation of intracystic floating balls is attributed to a “secretion-absorption imbalance,” where fluid from secretory epithelia is absorbed, concentrating insoluble sebum and keratin into spherules [7]. This process is complemented by the core aggregation hypothesis, where debris or hair shafts act as nuclei for the concentric layering of sebum and keratin [8].

For large abdominopelvic masses, CT is often the initial imaging modality of choice due to its wide Field of View (FOV), which facilitates comprehensive evaluation of the lesion and its relationship with adjacent structures [9]. However, CT has recognized limitations in determining the origin of large masses and differentiating lesions with overlapping imaging features [10]. This case highlights the diagnostic journey of a large MCT initially misdiagnosed on CT and emphasizes the critical role of a multimodal imaging strategy in reaching the correct diagnosis [11].

## 2 CASE PRESENTATION

### 2.1 Chief Complaints

A 40-year-old nulliparous female presented with a 3-month history of progressive, painless abdominal distension and recent urinary frequency/urgency.

### 2.2 Past Medical History

The patient reported no fever, dysuria, or weight loss. Menstrual cycles were regular, with no abnormal vaginal bleeding. She had no history of exposure to livestock, tropical animals, or pets, and no consumption of raw meat. Previous health check-ups (including blood and urine tests) were unremarkable, with no prior abdominal or pelvic ultrasound examinations.

### 2.3 Personal and Family History

The patient was a teacher with no history of chronic illnesses, surgeries, or hospitalizations. Family history was non-contributory, with no known genetic or endocrine disorders.

#### 2.3.1 Physical Examination

A soft, non-tender, palpable mass (15.0 × 20.0 cm) was noted at the umbilical level.

### 2.4 Laboratory Tests

Laboratory investigations revealed an elevated white blood cell count of 15.0 × 10^9^/L (normal range: 4-10 × 10^9^/L). Serum tumor markers were largely unremarkable, except for a mildly elevated Cancer Antigen 125 (CA-125) level of 45.0 U/mL (normal range: <35 U/mL). Preoperative parasite and larva identification via the direct smear method yielded negative results.

### 2.5 Imaging Examination

#### 2.5.1 CT

An abdominopelvic CT scan revealed a giant, thin-walled cystic mass measuring 17.5 × 9.5 × 21.8 cm. The cyst contained numerous hypodense spherules with thin walls (plain CT value < -5 HU), which had partial internal septa-like structures and few nodular calcifications (Fig. **1a**, **b**). These internal structures were initially interpreted as “daughter cysts”, raising suspicion for a hydatid cyst. Post-contrast scan revealed mild enhancement of the mass wall without internal enhancement or obvious fat density (Fig. **1c**). Adjacent organs were compressed, and ovarian structures were poorly visualized, making determination of the mass origin challenging.

### 2.6 Ultrasound

Grayscale imaging revealed a well-defined anechoic cyst with a maximum diameter of 22.4 cm in the right abdominopelvic cavity, containing multiple isoechoic spheres with internal linear hyperechoic strands (Fig. **2a**, **b**). Color Doppler imaging showed no internal vascularity within the spheres, and real-time transducer compression demonstrated the free-floating mobility of these spheres.

### 2.7 MRI

MRI identified a complex cystic-solid mass (17.2 × 8.2 × 22.4 cm) with well-defined margins originating from the right adnexa. The floating balls showed layered signal patterns: peripheral rim isointense on T1WI (T1-weighted imaging) and slightly hyperintense on T2WI, with a central core hypointense on T1WI and hyperintense on T2WI (Fig. **3a**, **b**). CE-T1WI FS (Contrast-Enhanced T1-Weighted Imaging with Fat Saturation) showed mild capsular enhancement. Crucially, suppression of the high T1 signal from the spheres was observed (Fig. **3c**, **d**), confirming their fat content. Diffusion-Weighted Imaging (DWI) and Apparent Diffusion Coefficient (ADC) imaging demonstrated restricted diffusion within the spherules (Fig. **3e**, **f**).

### 2.8 Intraoperative Findings

Exploratory laparotomy revealed a 25 cm right ovarian cystic-solid mass with an intact capsule, smooth surface, and good mobility (Fig. **4a**). Upon sectioning, the cyst was found to contain yellowish, sebaceous material, hair, and numerous free-floating spherical globules, consistent with the imaging findings (Fig. **4b**).

### 2.9 Pathological Diagnosis

Histopathological examination confirmed the diagnosis of a mature cystic teratoma, identifying tissues from all three germ layers: ectoderm (keratinized squamous epithelium, hair follicles), mesoderm (adipose tissue, cartilage), and endoderm (respiratory-type epithelium) (Fig. **5a**-**c**).

## 3 DISCUSSION

This case retrospectively describes an ovarian MCT initially misdiagnosed as a hydatid cyst on CT, and how a systematic multimodal approach, integrated with laboratory tests, ultimately clarified the diagnosis through recognition of the floating ball sign. While the floating ball sign itself is a known feature, this case's primary contribution lies in illustrating the diagnostic odyssey it prompted, serving as a paradigm for managing similar diagnostic conflicts.

Multimodal imaging played a pivotal role in reaching the correct diagnosis. Ultrasound effectively demonstrates the hyperechoic nature and crucial mobility of floating balls in real-time [12]. CT excels in quantifying fat content, with characteristic low attenuation values (-20 to -144 HU) being a key differentiator from water-density structures [5, 13]. MRI provides superior tissue characterization, with T1 hyperintensity and signal loss on fat-suppressed sequences confirming lipid composition [14-16].

Despite these characteristic findings, our case highlights the pitfall of misdiagnosis in CT. For massive abdominopelvic masses, CT is clinically favored as the initial imaging modality due to its wide Field Of View (FOV), which enables comprehensive evaluation of large lesions and their relationship with surrounding structures [9, 10, 17]. However, as this case demonstrates, this advantage can be offset by two key limitations when evaluating such large masses [18, 19].

First, the massive size of the lesion produced a significant mass effect, obscuring the adnexal structures and preventing the identification of its ovarian origin, a key clue for MCT. Second, the CT appearance of multiple internal spherules mimicked the classic “daughter cyst” sign of echinococcosis. CT’s limited soft-tissue contrast was insufficient to definitively distinguish the low-attenuation lipid content of the teratoma’s spheres from the water-density fluid of true daughter cysts. Additionally, CT failed to detect other hydatid-specific features such as obvious multiple calcified cyst walls or hyperattenuating cyst fluid (10–30 HU) [20], which differ from MCT’s fat-rich content (low attenuation, -20 to -144 HU) [5].

This CT-based suspicion, however, was inconsistent with the clinical picture. The patient lacked the typical epidemiological risk factors for hydatid disease, and crucially, echinococcal serology was negative. This discordance between imaging and laboratory findings was the primary impetus for further investigation.

The diagnostic dilemma was effectively resolved by subsequent imaging. With ultrasound and Magnetic Resonance Imaging (MRI), despite their narrower FOV compared to CT, these limitations are offset by their superior tissue-specificity. The real-time dynamic capability of ultrasound played a pivotal role by demonstrating the mobility and floatability of intracystic spherical structures, a feature not seen in hydatid cysts, where internal structures are typically fixed or represent detached membranes [12, 18]. This finding fundamentally challenged the initial CT diagnosis.

MRI then provided definitive tissue characterization. The key diagnostic sequence was fat suppression, which caused a marked signal drop in the T1-hyperintense spheres, unequivocally confirming their fat composition [14-16]. The absence of T2-hypointense septa (typical of hydatid cysts) further supported the diagnosis of MCT.

Based on this experience, when a conflict arises between CT findings and clinical context, we propose a deliberate “diagnostic reconsideration pathway, which is as follows:

(1) Re-evaluate the initial CT for subtle signs such as fat attenuation (HU < -20) that may have been overlooked [13].

(2) Perform targeted, dynamic ultrasound to assess for mobility of internal structures, a feature that effectively distinguishes MCT from hydatid disease [12, 18].

(3) Utilize MRI with fat-saturation sequences as a definitive problem-solving tool to confirm lipid composition [14-16].

This stepwise approach transforms potential misdiagnosis into a confirmed diagnosis, leveraging the complementary strengths of each modality while acknowledging their individual limitations.

Beyond hydatid disease, the differential diagnosis for a large, complex cystic ovarian mass includes serous or mucinous cystadenomas and hemorrhagic cysts. While cystic ovarian neoplasms such as cystadenocarcinoma or metastatic tumors may present as complex cystic masses, the absence of solid enhancing components and the presence of macroscopic fat effectively exclude these malignancies. In resource-limited settings, ultrasound demonstration of mobile, hyperechoic spheres with the “dot-dash” sign from hair can be highly suggestive of MCT even without MRI [10, 11]. However, the presence of macroscopic fat, as confirmed by MRI, and the distinct, mobile spherical morphology of the internal contents effectively excluded these alternatives [11, 21, 22]. Key differentiating imaging features of the main possible diagnoses are summarized in Table **1**.

Management of MCTs is surgical. While laparoscopic cystectomy is preferred for fertility preservation, laparotomy was appropriately chosen in this case due to the immense size of the mass, which posed a risk of rupture and spillage during a minimally invasive approach. Intraoperative findings of sebaceous material, hair, and floating balls strongly support the preoperative diagnosis. Frozen section analysis should be considered if features suggestive of immaturity (large solid components, necrosis) or malignant transformation are present. Despite the excellent long-term prognosis and low recurrence rates (1-2%) associated with MCTs, postoperative surveillance with ultrasonography is still recommended to monitor for these rare events [22, 23].

## CONCLUSION

The floating ball sign is a key diagnostic feature of ovarian MCTs, but as this case demonstrates, its appearance on CT alone can be misleading. In large abdominal-pelvic cystic masses where CT findings are ambiguous or conflict with the clinical context, targeted ultrasound to assess internal mobility and MRI with fat-saturation sequences are critical next steps. This systematic multimodal imaging approach is essential for avoiding misdiagnosis and guiding appropriate surgical management.

## PATIENT PERSPECTIVE

**The Patient’s Perspective was as Follows**

“When I noticed my abdomen gradually enlarging, I initially thought it was weight gain. It was not until I developed frequent urination that I sought medical attention. The initial CT suggestion of a hydatid cyst frightened me, especially with the uncertainty before further tests. I was relieved when the doctors performed additional ultrasound and MRI, carefully explaining that they found characteristic 'floating balls' pointing to a benign condition.”

“The surgery went smoothly, and my recovery was uneventful. I am grateful for the thorough diagnostic approach that prevented misdiagnosis and unnecessary anxiety. I hope sharing my experience helps other patients facing similar diagnostic uncertainty.

## Figures and Tables

**Fig. (1):**
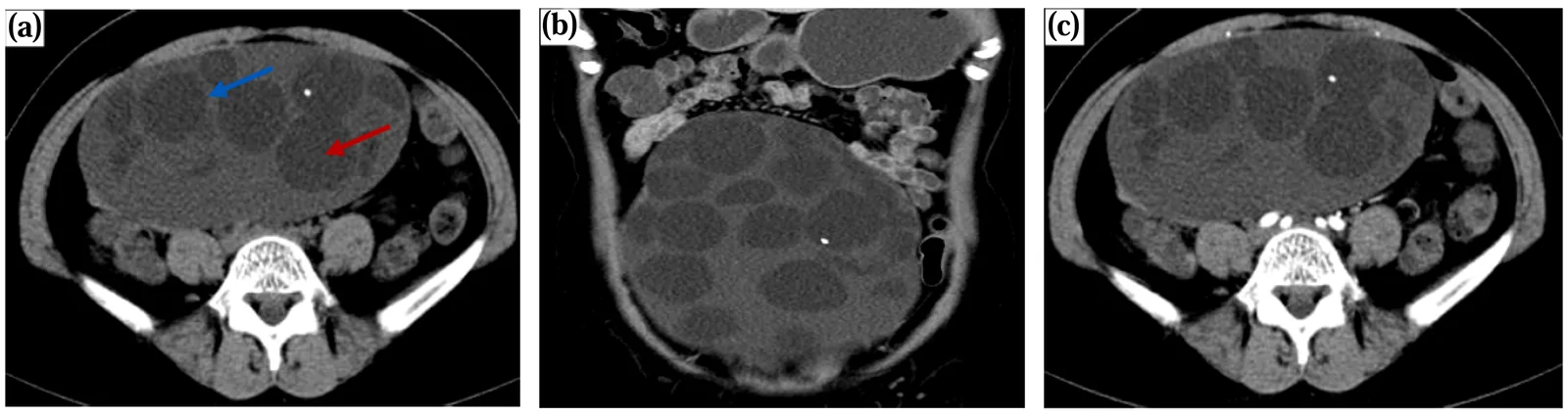
Abdominopelvic CT findings. (**a**, **b**) Axial and sagittal plain CT images demonstrate a giant, thin-walled cystic mass measuring 17.5 × 9.5 × 21.8 cm. Multiple hypodense balls with thin walls (**red arrows**) and internal septa-like structures (**blue arrows**) are visible within the cyst. The measured attenuation of the balls was < -5 HU. (**c**) Post-contrast CT scan reveals mild enhancement of the mass wall without internal enhancement.

**Fig. (2):**
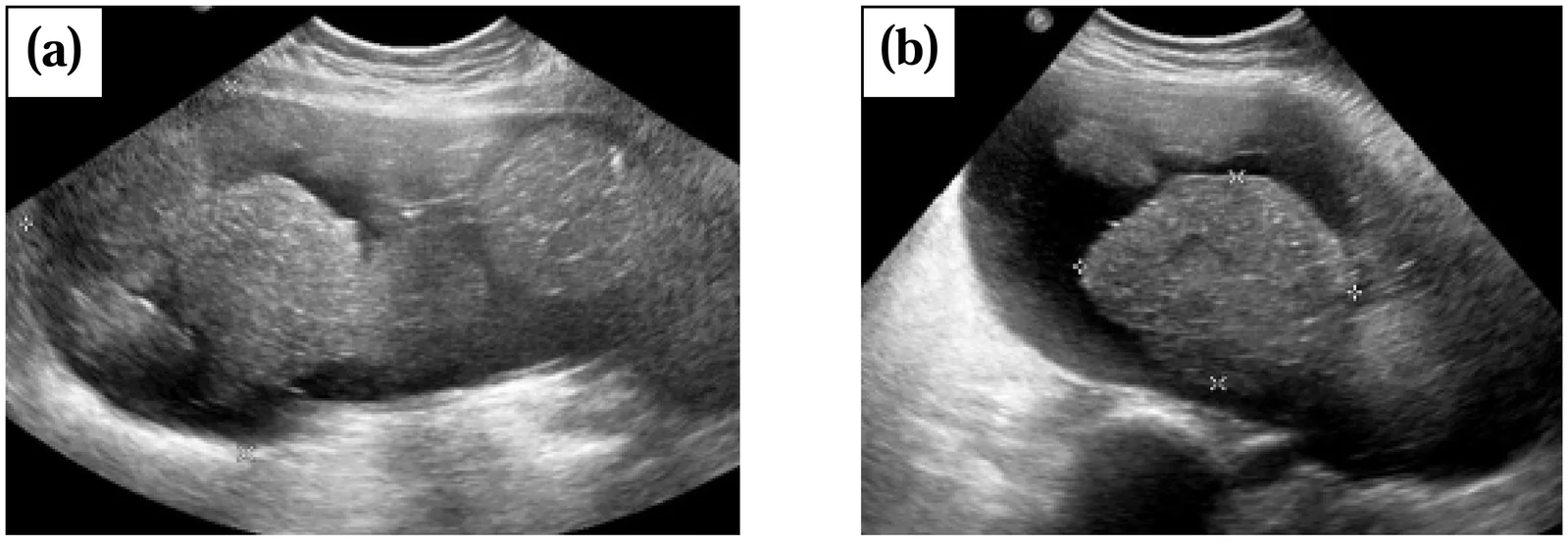
Abdominopelvic Ultrasound findings. (**a**) A well-defined anechoic cyst containing multiple isoechoic spheres. (**b**) Detailed view of a single isoechoic sphere (calipers) demonstrating its smooth contour and internal linear hyperechoic strands.

**Fig. (3):**
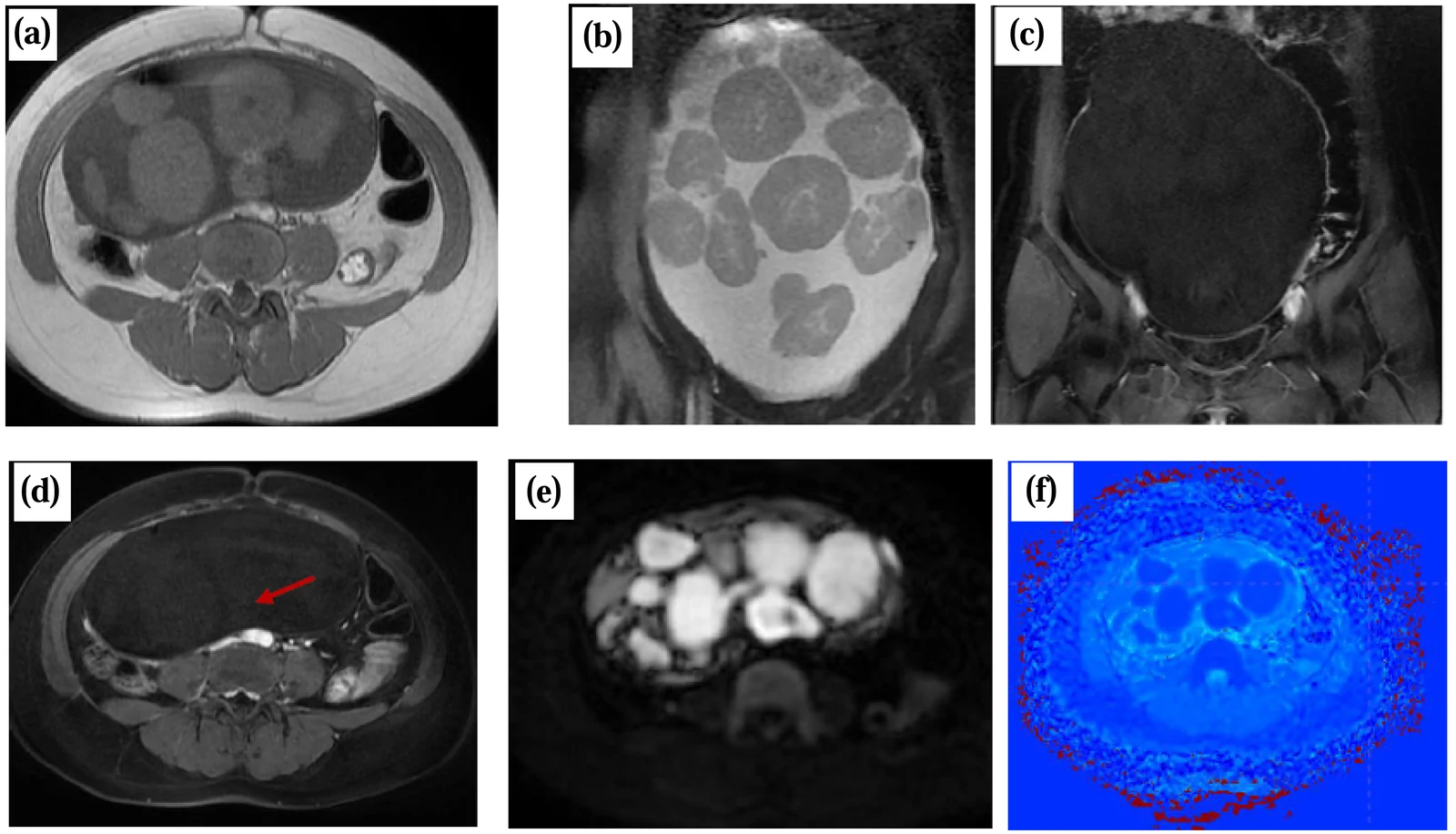
Abdominopelvic MRI findings. (**a**-**b**) T1WI and T2WI show layered signal patterns of the balls. (**c**-**d**) CE-T1WI FS shows mild capsular enhancement and suppression of high T1 signal (**red arrow** in d), confirming fat content. (**e**-**f**) DWI and color ADC demonstrate restricted diffusion within the spherules (low ADC values in dark blue).

**Fig. (4):**
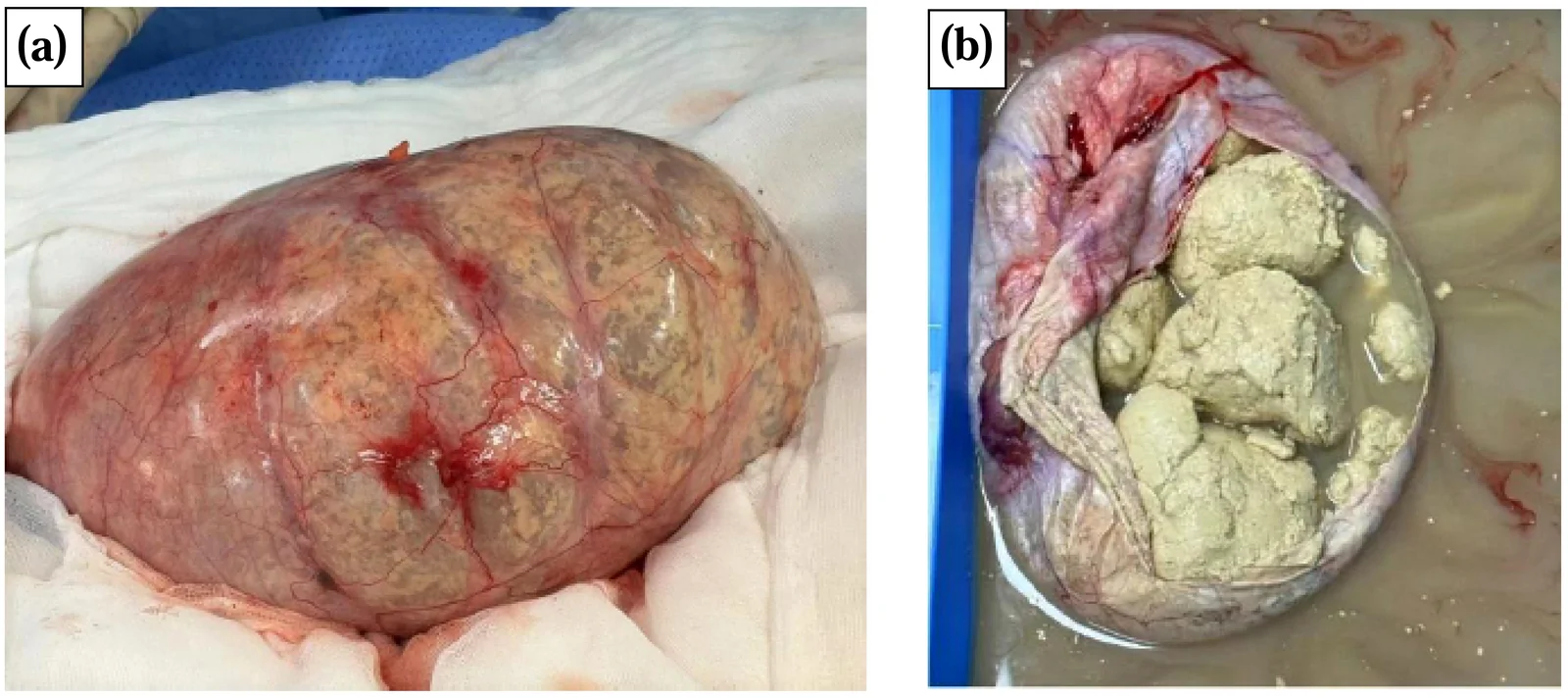
Intraoperative findings. (**a**) A 25 cm right ovarian cystic-solid mass. (**b**) Upon sectioning, the cyst contained sebaceous material, hair, and free-floating spherical globules.

**Fig. (5):**
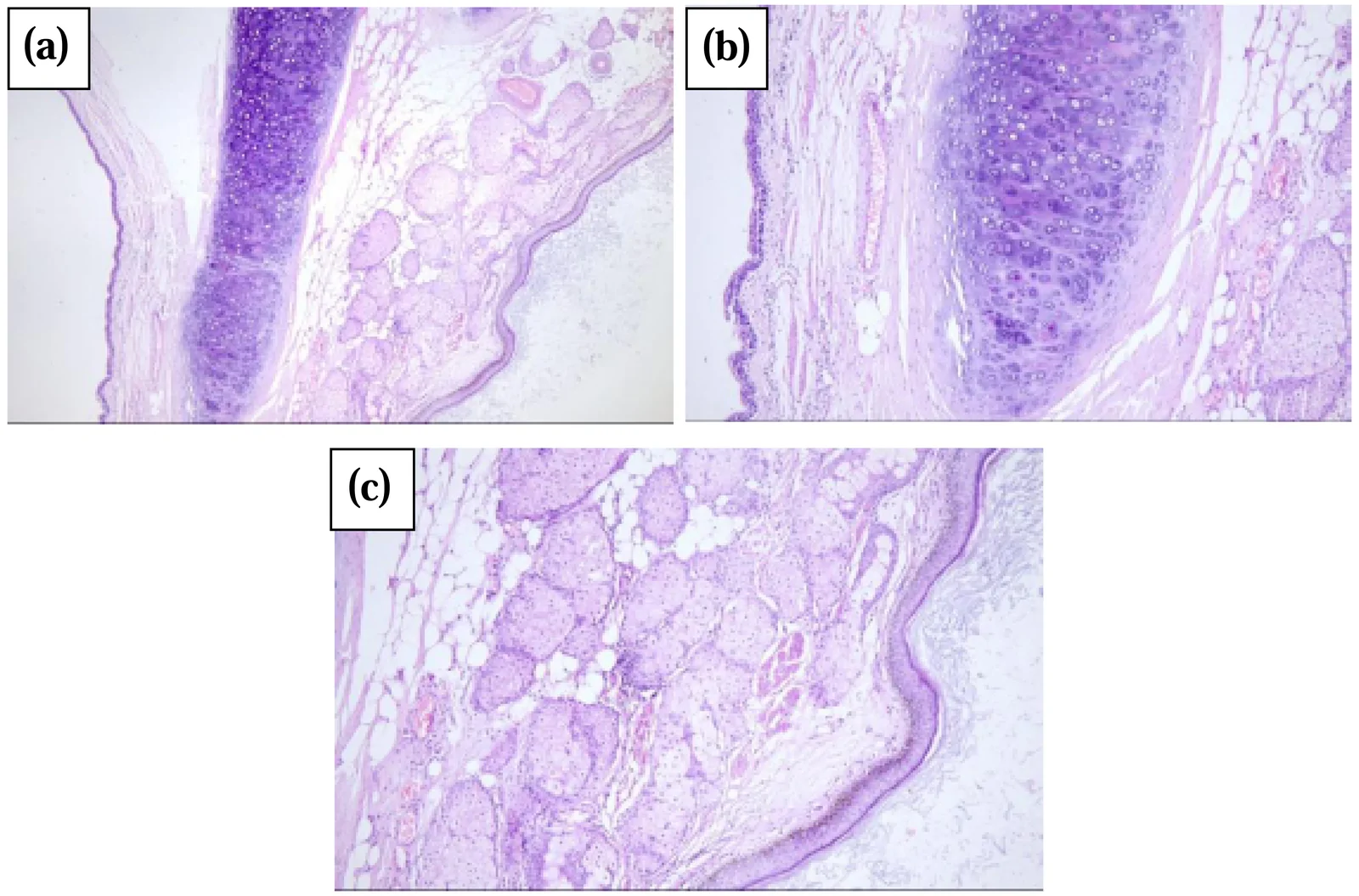
Pathological findings. (**a**) Squamous epithelium and skin appendages (ectoderm, right), cartilage (mesoderm, middle), respiratory tract epithelium (endoderm, left). (**b**) Magnification of squamous epithelium and cutaneous appendages. (**c**) Magnification of the cartilage (right) and respiratory tract epithelium (left).

**Table 1:** Key differentiating features of giant ovarian cystic masses on multimodal imaging.

**Pathology**	**CT Findings**	**Ultrasound Findings**	**MRI Findings**	**Key Feature**
**Mature Cystic Teratoma**	Fat-containing lesion (-20 to -144 HU) with floating balls; fat-fluid level; calcifications [5]	Anechoic cyst with mobile hyperechoic spheres; “dot-dash” sign from hair; posterior acoustic shadowing [12]	T1-hyperintense spheres with signal loss on fat-saturation; variable DWI restriction [13, 16]	Mobile, fat-containing spheres (Floating Ball Sign)
**Hydatid Cyst (Echinococcosis)**	Well-defined cyst with “daughter cysts” (water density, 0-20 HU); membrane detachment; wall calcifications [18, 20]	Cyst with fixed internal septations (“wheel-like”); “sandstorm” sign from debris [18]	“Daughter cysts” with a hypointense rim on T2WI; no fat suppression [19]	Non-mobile internal structures (Daughter cysts)
**Serous/Mucinous Cystadenoma**	Large, thin walled, unilocular or multilocular cyst; fluid density (0-20 HU); thin septa: no fat[10]	Anechoic or with fine internal septations; no internal mobility; no solid components [10]	Simple fluid signal (T1 low, T2 high); thin enhancing septa; no fat [10]	Simple cystic structure without solid or fat components

## Data Availability

The data of current study are available from corresponding author, [Y.H], on a reasonable request.

## LIST OF ABBREVIATIONS

ADC: Apparent Diffusion Coefficient
CA-125: Cancer Antigen 125
CT: Computed Tomography
CE-T1WI FS: Contrast-Enhanced T1-Weighted Imaging with Fat Saturation
DWI: Diffusion-Weighted Imaging
FOV: Field Of View
HU: Hounsfield Units
MCT: Mature Cystic Teratoma
MRI: Magnetic Resonance Imaging
T1WI: T1-Weighted Imaging
T2WI: T2-Weighted Imaging

## STANDARDS OF REPORTING

CARE guidelines were followed.
